# Screening and Evaluation of Rice to Assess Antibiosis and Antixenosis Resistance to White-Backed Planthopper (*Sogatella furcifera*)

**DOI:** 10.3390/plants15050811

**Published:** 2026-03-06

**Authors:** Jariya Roddee, Kamolchanok Umnajkitikorn, Napatson Chansawang, Jirapong Jairin, Jureemart Wangkeeree

**Affiliations:** 1 School of Crop Production Technology, Institute of Agricultural Technology, Suranaree University of Technology, Nakhon Ratchasima 30000, Thailand; k.umnajkitikorn@g.sut.ac.th (K.U.); 123tiptib@gmail.com (N.C.); 2 Ubon Ratchathani Rice Research Center, Muang, Ubon Ratchathani 34000, Thailand; jjairin@gmail.com; 3 Department of Agricultural Technology, Faculty of Science and Technology, Thammasat University Rangsit Centre, Pathum Thani 10200, Thailand; jureemart@yahoo.com

**Keywords:** *Sogatella furcifera*, feeding behavior, physiological responses, gene expression

## Abstract

The white-backed planthopper, *Sogatella furcifera*, and the brown planthopper, *Nilaparvata lugens*, severely impact rice production, necessitating effective selection methods for resistant cultivars. *S. furcifera* poses a significant threat to rice cultivation, particularly in Asia. Through this study, we aimed to establish an effective approach to identifying resistant rice varieties based on feeding behavior, physiological and chemical responses, and genetic analysis. Three key activities were involved: (1) evaluation of planthopper feeding behavior utilizing the honeydew drop method, the electrical penetration graph technique, and growth rate analysis; (2) investigation into the physiological and chemical traits of rice; and (3) analysis of resistance-related gene expression. The results indicated larger honeydew drop areas, fewer and shorter probing events, and structural defenses such as increased trichome density in resistant rice genotypes, likely hindering insect attachment and feeding. We confirmed the suitability of the growth rate method for resistance screening. Gene expression analysis identified *PR10a* upregulation in resistant rice, suggesting a molecular basis for resistance. This study enables the selection of rice varieties resistant to planthoppers, supporting sustainable pest management and breeding programs. The findings support sustainable pest management by enabling the targeted selection of resistant varieties, ultimately aiding in the development of rice genotypes with enhanced resistance across growth stages.

## 1. Introduction

Rice (*Oryza sativa* L.) underpins global food security; however, recurrent outbreaks of planthoppers—principally the brown planthopper (*Nilaparvata lugens* Stål; BPH) and the white-backed planthopper (*Sogatella furcifera* Horváth; WBPH)—continue to impact yield stability across South, Southeast, and East Asia. Beyond direct phloem-feeding injuries (hopperburns), these vectors transmit rice ragged stunt virus (RRSV) and rice grassy stunt virus (RGSV), intensifying the risks to production systems already under stress from climate variability and intensive cultivation (e.g., high-nitrogen monocultures) [1]. Rice production is persistently impacted by sap-sucking insect pests, particularly planthoppers (Hemiptera: Delphacidae). Among these, the WBPH is one of the most destructive pests, causing severe yield losses through direct phloem ingestion and disruption of plant physiological processes [2,3]. Frequent WBPH outbreaks have been reported across South and Southeast Asia, often associated with shifting cultivation systems and mismanagement of insecticides [4].

Chemical insecticides remain the primary strategy for WBPH control. However, their intensive and repeated use has led to the development of resistance, pest resurgence, and adverse effects on non-target organisms and the environment [5,6]. Consequently, host plant resistance is widely recognized as a sustainable and environmentally sound approach to long-term planthopper management [7]. In rice, planthopper resistance is typically categorized into three mechanisms: antibiosis, antixenosis, and tolerance [8]. Among these, antibiosis and antixenosis play central roles in limiting planthopper feeding success, development, and host selection behavior and are therefore the primary focus of resistance breeding programs. Antibiosis resistance directly affects the insect’s biology by reducing survival, growth, or reproduction after feeding on resistant plants [8,9]. In rice–planthopper interactions, antibiosis is often associated with impaired phloem sap ingestion, reduced feeding efficiency, and delayed insect development [10]. These effects can be quantified using feeding-related parameters, such as honeydew excretion [11], insect growth rate, and detailed analysis of feeding behavior. A choice test is utilized to select plant varieties resistant to insect pests. This practical method for observing the non-preference behavior of insect pests toward host plants involves many factors, such as signals and information content, volatile compounds specific to individual host plants, and sensory aspects, enabling insects to receive signals from the host [12]. In contrast, antixenosis resistance influences insect behavior through deterring host acceptance, settling, or sustained feeding [8,13]. Morphological traits, including trichome density and leaf sheath surface characteristics, may create physical barriers that impede insect attachment and probing, thereby discouraging feeding initiation [14].

Advances in plant–insect interaction research have highlighted the importance of comprehensive screening approaches that combine behavioral, physiological, biochemical, and molecular analyses. The honeydew excretion assay is a rapid and effective method for estimating phloem ingestion efficiency in planthoppers [15]. The electrical penetration graph (EPG) technique provides detailed insights into stylet penetration, probing frequency, and phloem sap ingestion, allowing discrimination between resistant and susceptible host plants at a fine behavioral scale [16,17,18,19,20,21,22,23]. In addition, insect growth rate assays offer a robust indicator of antibiosis, directly reflecting host plant effects on insect development and fitness [10,17]. At the plant level, resistance to planthoppers is frequently associated with structural defenses, altered phytohormone signaling, and the activation of defense-related genes [24]. Pathogenesis-related (PR) proteins, particularly *PR10*, have been associated with rice defense responses against sap-sucking insects and may contribute to antibiosis by impairing insect feeding and development [25,26,27]. The integration of phenotypic resistance traits with molecular markers such as *PR10a* expression can therefore enhance resistance screening and facilitate breeding for durable resistance.

Despite the economic importance of WBPH in Thailand, comprehensive screening frameworks that integrate feeding behavior, plant physiological traits, and molecular resistance indicators remain limited. Previous studies have primarily focused on BPH resistance or relied on single evaluation methods, which often fail to capture the complex WBPH–rice interactions [7,8]. Therefore, in this study, we aimed to establish an integrated approach to identifying WBPH-resistant rice genotypes by combining feeding-behavior analysis (honeydew excretion, EPG, and insect growth rate), evaluation of plant morphological and chemical traits, and expression analysis of resistance-related genes. By linking antibiosis and antixenosis mechanisms with multi-level phenotypic and molecular indicators, this work provides a robust framework for WBPH resistance screening, supporting sustainable rice breeding and pest management programs.

## 2. Results

### 2.1. Honeydew Excretion and Salivary Sheath Formation Assay

**Figure 1:**
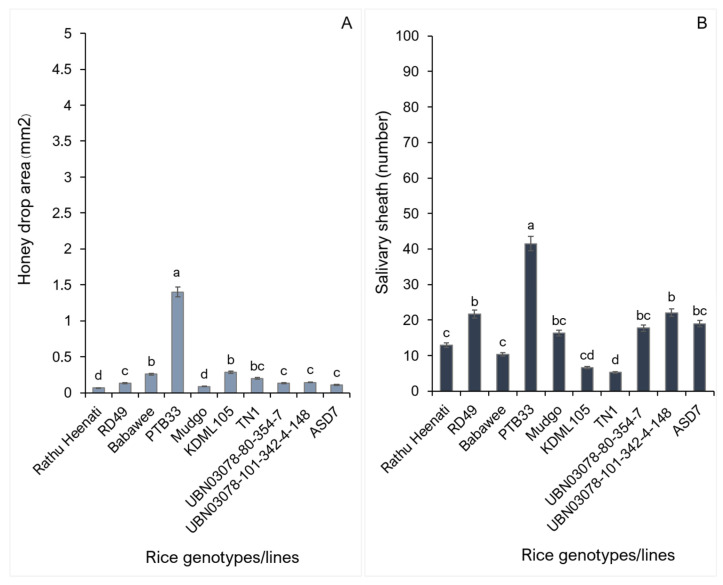
Feeding activity of the white-backed planthopper on 10 rice genotypes: (**A**) honeydew area excreted after 24 h and (**B**) number of salivary sheaths. Bars indicate mean ± SE (*n* = 20). Different letters indicate significant differences among genotypes (one-way ANOVA, Tukey’s HSD, *p* < 0.05).

The feeding intensity of the WBPH on 10 rice genotypes/lines was quantified using honeydew excretion and salivary sheath formation after a 24 h feeding (Figure 1). Differences in honeydew excretion were significant among the rice genotypes (Figure 1A) (F_9194_ = 1.03, *p* = 0.041). The PTB33 showed the largest honeydew area (mean 1.4 cm^2^), significantly exceeding other genotypes. Honeydew excretion was intermediate on Babawee and KDML105, whereas TN1, UBN03078-80-354-7, UBN03078-101-342-4-148, and ASD7 demonstrated relatively low honeydew areas. Honeydew excretion was lowest in Rathu Heenati and Mudgo, indicating reduced WBPH feeding activity in these genotypes (Figure S1).

The salivary sheath numbers are shown in Figure 1B. WBPH feeding on PTB33 resulted in the highest frequency of salivary sheaths (>40), significantly exceeding the remaining genotypes (F_9197_ = 9.23, *p* < 0.001). In contrast, Rathu Heenati, Babawee, and TN1 supported significantly fewer salivary sheaths, reflecting limited probing and feeding behavior. Genotypes characterized by low honeydew excretion generally corresponded to lower salivary sheath numbers.

### 2.2. Electrical Penetration Graph Study

#### 2.2.1. Feeding Behavior of White-Backed Planthopper (WBPH) on Different Rice Genotypes

EPG analysis revealed seven distinct waveform patterns associated with WBPH probing and feeding behavior, as illustrated. These waveforms were classified as Np, N1-N2, N3, N4-a, N4-b, and N5, and their characteristics were consistent with previously described feeding activities [19,20,28].

#### 2.2.2. Number of Waveform Events per Insect (NEWI)

**Figure 2:**
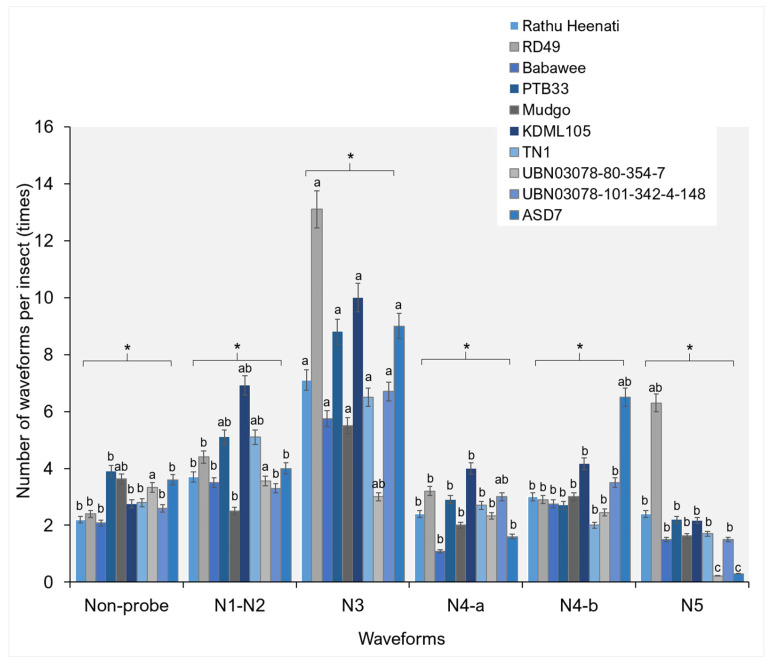
Number of waveform events per insect in the white-backed planthopper on different rice genotypes. Bars (mean ± SEM) with the same letter at the top within a waveform category are not significantly different at *p* = 0.05 (Tukey’s test). Np = non-probe; N1, N2 = Pathway phase; N3 = pathway in epidermis/mesophyll; N4-a = phloem salivation or sieve-element salivation; N4-b = sustained phloem sap ingestion or phloem ingestion; and N5 = xylem ingestion. An asterisk (*) in a figure indicates a statistically significant difference between groups, where the *p*-value < 0.05.

The NEWI differed significantly among rice genotypes across all EPG waveform categories (Figure 2), indicating genotype-dependent effects on the insect’s probing and feeding behavior. Non-probing (Np) events were generally low across genotypes, with TN1 and Rathu demonstrating significantly higher frequencies than PTB33 and Babawee (F_9292_ = 1.49, *p* = 0.016). The frequency of pathway-phase waveforms (N1–N2) varied markedly among genotypes, with PTB33 and Mudgo indicating significantly higher NEWI values than Rathu Heenati, RD49, and Babawee (F_9420_ = 2.16, *p* = 0.034).

Significant differences were also observed during the phloem contact phase (N3). The genotype RD49 demonstrated the highest frequency of N3 events, followed by KDML105 and PTB33, whereas genotypes such as Mudgo indicated significantly fewer N3 events (F_9754_ = 1.90, *p* = 0.0041). For phloem sap ingestion waveforms, both N4-a and N4-b phases demonstrated clear genotype-specific patterns. In particular, KDML105 and ASD7 indicated significantly higher N4-a (F_9252_ = 3.73, *p* = 0.005) and N4-b (F_9329_ = 3.17, *p* = 0.002), respectively. Xylem ingestion events (N5) occurred at low frequencies across all genotypes; however, RD49 varieties demonstrated significantly higher NEWI values than other genotypes/lines (F_9199_ = 5.01, *p* < 0.001) (Figure 2).

#### 2.2.3. Waveform Duration per Event per Insect (WDEI)

**Figure 3:**
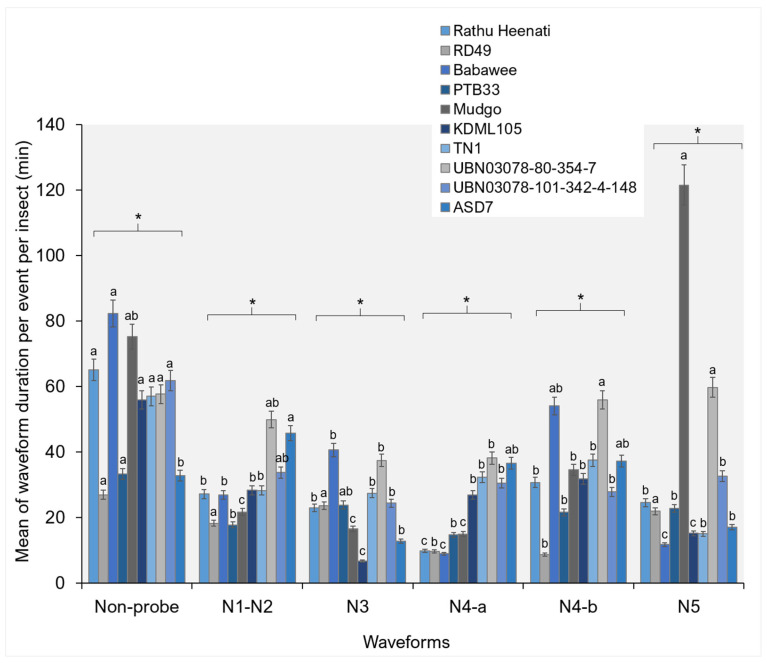
The waveform duration per event per insect in the white-backed planthopper on different rice genotypes. Bars (mean ± SEM) with the same letter at the top within a waveform category are not significantly different at *p* = 0.05 (Tukey’s test). Np = non-probe; N1, N2 = pathway phase; N3 = pathway in epidermis/mesophyll; N4-a = phloem salivation or sieve-element salivation; N4-b = sustained phloem sap ingestion or phloem ingestion; and N5 = xylem ingestion. An asterisk (*) in a figure indicates a statistically significant difference between groups, where the *p*-value < 0.05.

WDEI differed significantly among rice genotypes across all EPG waveform categories (Figure 3). In the NP phase, genotypes, particularly RD49, PTB33, TN1, and Rathu, showed significantly longer waveform durations than Babawee and Mudgo (F_9292_ = 2.17, *p* = 0.041), suggesting prolonged searching or resting behavior before stylet insertion. During the pathway phase (N1–N2), significant genotype-dependent variation was detected (F_9420_ = 3.57, *p* = 0.048). UBN03078-80-354-7 and ASD7 genotypes demonstrated prolonged event durations compared to other genotypes, indicating increased difficulty in stylet penetration through plant tissues. Significant differences were also observed in the N3 waveform duration (F_9754_ = 4.65, *p* = 0.037). In particular, Babawee and UBN03078-80-354-7 PTB33 demonstrated significantly longer N3 durations than KDML105 genotypes. For phloem-related feeding, the durations of the N4-a and N4-b waveforms differed significantly among genotypes. UBN03078-80-354-7 genotypes demonstrated significantly longer N4-a (F_9252_ = 1.94, *p* = 0.042) and N4-b (F_9329_ = 2.47, *p* = 0.041), indicating sustained phloem sap ingestion. In contrast, resistant genotypes demonstrated markedly reduced phloem-feeding durations, indicating impaired feeding efficiency. Xylem ingestion events (N5) also showed significant variation among genotypes (F_9199_ = 7.24, *p* = 0.021), with the Mudgo and UBN03078-80-354-7 genotypes exhibiting prolonged N5 durations compared to other lines (Figure 3).

#### 2.2.4. Waveform Duration per Insect (WDI)

**Figure 4:**
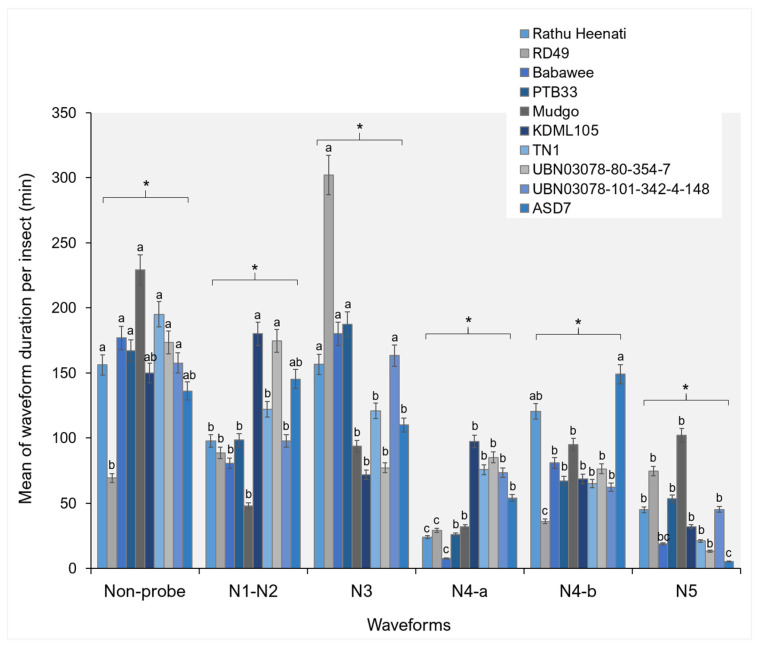
The WDI of the white-backed planthopper on different rice genotypes. Bars (mean ± SEM) with the same letter at the top within a waveform category are not significantly different at *p* = 0.05 (Tukey’s test). Np = non-probe; N1, N2 = pathway phase; N3 = pathway in epidermis/mesophyll; N4-a = phloem salivation or sieve-element salivation; N4-b = sustained phloem sap ingestion or phloem ingestion; and N5 = xylem ingestion. An asterisk (*) in a figure indicates a statistically significant difference between groups, where the *p*-value < 0.05.

WDI differed significantly among rice genotypes for all EPG waveform categories (Figure 4). The Np phase indicated that the RD49 and PTB33 genotypes had significantly shorter cumulative durations than the other genotypes (F_9292_ = 0.64, *p* = 0.047). In particular, Mudgo and Babawee demonstrated the longest NP durations. During the pathway phase (N1–N2), significant genotype-dependent variation in WDI was observed (F_9420_ = 1.57, *p* = 0.041). ASD7 and UBN03078-80-354-7 genotypes exhibited longer pathway durations than RD49 and PTB33 genotypes. Significant differences were also detected in the N3 waveform duration, with KDML105 and Mudgo indicating shorter durations (F_9754_ = 1.53, *p* = 0.014). For phloem-related feeding phases, both N4-a waveform durations differed significantly among rice genotypes (F_9252_ = 4.57, *p* < 0.001). TN1, UBN03078-80-354-7, UBN03078-101-342-4-148, and ASD4 genotypes demonstrated significantly extended cumulative durations of phloem ingestion. Similarly, N4-b waveform durations in UBN03078-80-354-7 genotypes were prolonged, indicating impaired sustained phloem sap ingestion (F_9329_ = 1.84, *p* = 0.043). Xylem ingestion (N5) durations also varied significantly among genotypes (F_9199_ = 8.40, *p* = 0.005), with Mudgo genotypes demonstrating prolonged N5 durations compared to other genotypes.

### 2.3. WBPH Survival and Development Bioassays on Different Rice Genotypes

**Figure 5:**
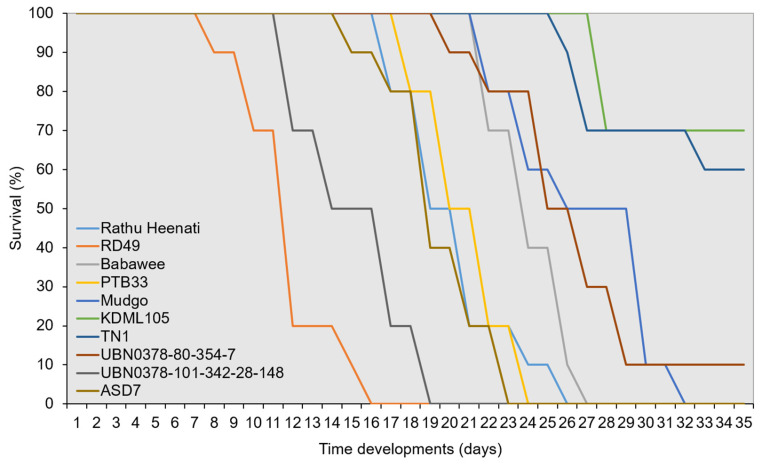
Survival dynamics of the white-backed planthopper (*Sogatella furcifera*) across developmental stages on 10 rice genotypes.

The nymphal development and survival of the WBPH differed markedly among the 10 rice genotypes evaluated at the seedling stage (Figure 5). Survival remained at 100% during the egg stage and the first- and second-instar stages across all genotypes, indicating no early-stage mortality. From the third instar onward, genotype-dependent divergence was evident in survival trajectories. Kaplan–Meier survival analysis demonstrated significant differences in cumulative survival probabilities among rice genotypes (χ^2^ = 5.42, df = 9, *N* = 199, *p* < 0.001). The ASD7, Babawee, and Rathu Heenati genotypes exhibited a rapid decline in survival from the third-to-fourth-instar stage onward. Survival on TN1 decreased to approximately 50% at adult emergence (15.26 days). In contrast, the TN1 and KDML105 genotypes demonstrated significantly higher survival probabilities throughout nymphal development. Kaplan–Meier curves for these genotypes indicated minimal mortality during mid- and late-nymphal stages, with survival exceeding 80–90% at adult emergence. Intermediate survival patterns were observed in the Babawee and UBN03078-80-354-7 lines, which demonstrated gradual declines in survival, primarily during the fourth- and fifth-instar stages (24.43 and 17.14 days), resulting in adult emergence rates of approximately 70–75%. RD49 lines demonstrated low survival in the fifth-instar stage and failed to develop to the adult stage. In contrast, the RD49 line showed markedly reduced survival at the fifth instar, and no individuals survived to the adult stage.

The developmental duration of WBPH varied significantly among the 10 rice genotypes evaluated (Table 1). Significant genotype effects were detected at the first-instar, third-instar, and adult stages (*p* ≤ 0.05), whereas no significant differences were observed during the second-, fourth-, and fifth-instar stages. In the first-instar stage, nymphs reared on Babawee and UBN03078-80-354-7 demonstrated extended developmental durations compared to those reared on KDML105, TN1, UBN03078-101-342-4-148, and Rathu Heenati, which demonstrated significantly shorter durations (1 ± 0.0 days, *p* ≤ 0.05). During the third instar, development was significantly prolonged on PTB33 (mean 3.30 ± 1.38 days) and Rathu Heenati (mean 3.25 ± 1.47 days). Nymphs on UBN03078-80-354-7 and Mudgo completed this stage more rapidly (1.60 ± 0.83 and 1.05 ± 0.59, *p* ≤ 0.05, respectively). Adult longevity also differed significantly among rice genotypes, with a mean of 12.83 ± 4.29 days. The longest adult duration was observed on Mudgo (12.83 ± 4.29 days), followed by Babawee (9.24 ± 0.83 days) and KDML105 (8.86 ± 2.71 days), whereas substantially shorter adult durations were observed on RD49 and UBN03078-101-342-148.

### 2.4. Trichome Density

**Figure 6:**
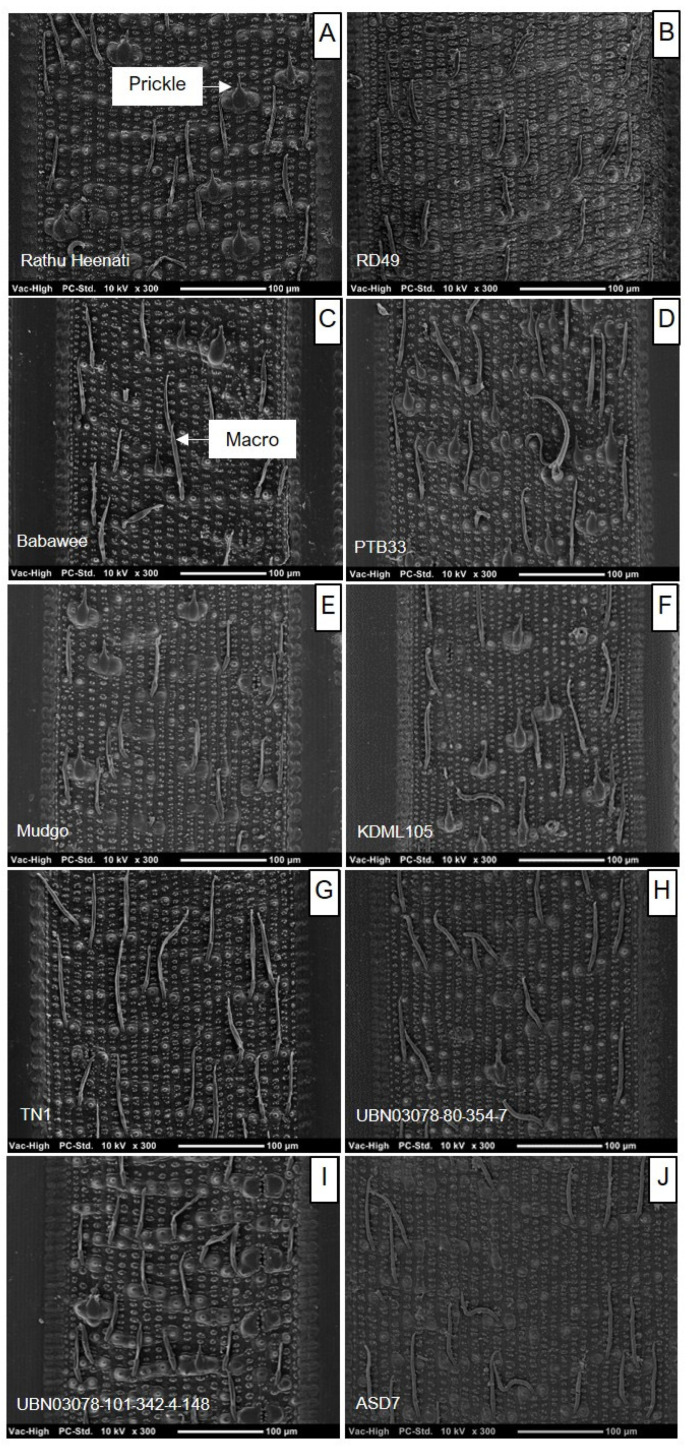
Trichome of abaxial leaf surface for 10 rice genotypes using scanning electron micrography: (**A**) Rathu Heenati, (**B**) RD49, (**C**) Babawee, (**D**) PTB33, (**E**) Mudgo, (**F**) KDML105, (**G**) TN1, (**H**) UBN03078-80-354-7, (**I**) UBN03078-101-342-148, (**J**) ASD7. SEM of abaxial leaf surface at 0.3 mm^2^.

**Figure 7:**
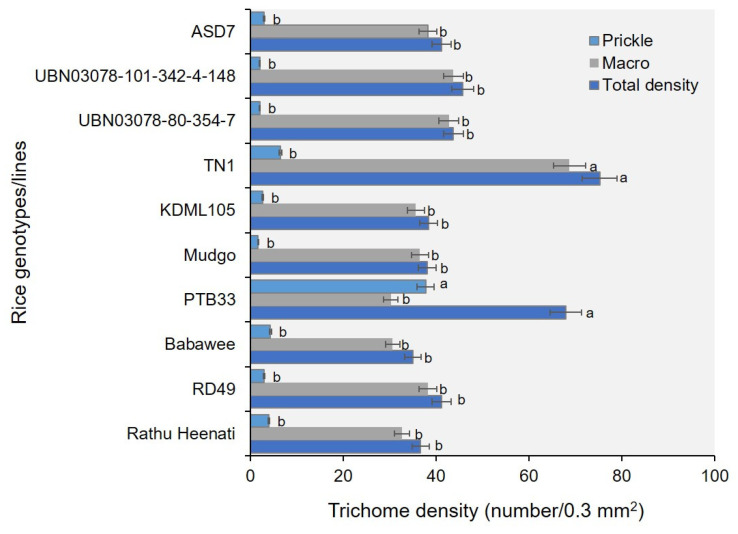
Comparison of trichome density on the abaxial leaf surface among rice genotypes. Bars (mean ± SEM) with the same letter at the top within a waveform category are not significantly different according to Tukey’s honestly significant difference (HSD) test (*p* = 0.05).

Scanning electron microscopy (SEM) revealed significant variation in trichome density on the abaxial surface of leaves among the ten genotypes investigated (Figure 6). All plant trichomes were found in this non-granular trichome type. The TN1 demonstrated the highest macrotrichome and total trichome densities, significantly greater than those of all other genotypes (F_989_ = 1.84, *p* < 0.050) (Figure 7). Similarly, PTB33 showed high total trichome density. In contrast, the ASD7, Mudgo, KDML105, RD49, Babawee, and Rathu Heenati genotypes showed significantly lower macrotrichome and total trichome densities, with no significant differences among them (*p* > 0.05).

### 2.5. Correlation and Principal Component Analysis Between the Parameters

**Figure 8:**
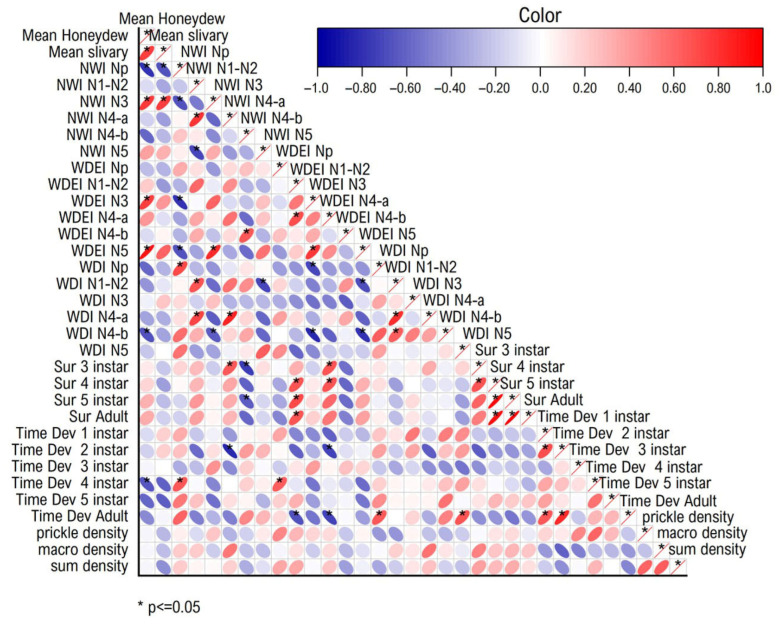
Correlation coefficients (Pearson correlation) and significance levels of honeydew excretion, salivary sheath, EPG analysis for white-backed planthopper’s (*Sogatella Furcifera*) feeding behavior, development, and survival rate, and trichome factors. Red circles indicate positive correlations, whereas blue circles indicate negative correlations. The shape and narrowness of the ellipses reflect the strength of the correlation, with narrower ellipses representing stronger correlations approaching ±1 and more circular shapes indicating weaker relationships. The color intensity corresponds to the magnitude of the correlation coefficient, ranging from −1 (strong negative correlation) to +1 (strong positive correlation). Asterisks (*) denote statistically significant correlations at *p* ≤ 0.05.

The correlation analysis conducted in this study was designed to explore the relationships among honeydew excretion, salivary sheath, EPG analysis for WBPH feeding behavior, development and survival rate, and trichome factors (Figure 8). Conversely, the mean honeydew value was positively correlated with WDI N4 (*r* = 0.93 *) and WDEI N5 (*r* = 0.91 *) and with NWI N3, WDEI N3, salivary sheath, and WDI N1N2 (*r* = 0.78 *, *r* = 0.75 *, *r* = 0.74, and *r* = 0.73, respectively). Additionally, negative correlations were observed between mean honeydew and specific EPG waveform parameters, such as NWI NP (*r* = −0.79 *). Additionally, the salivary sheath was positively correlated with NWI N3 (*r* = 0.75 *) but negatively correlated with NEI NP (*r* = −0.79 *).

**Figure 9:**
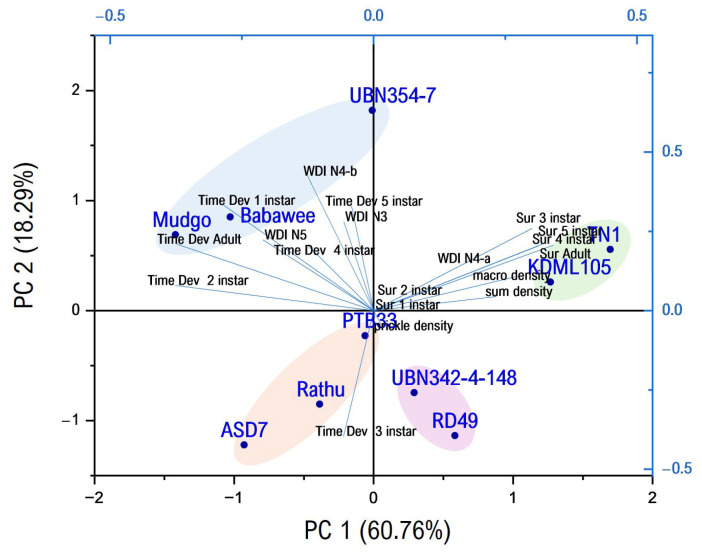
Principal component analysis (PC1 and PC2) of 10 rice genotypes based on honeydew excretion, salivary sheath, EPG analysis for WBPH’s (*Sogatella Furcifera)* feeding behavior, development, and survival rate, and trichome factors.

Principal component analysis (PCA) demonstrated that the first two PCs together accounted for 79.05% of the total variance, with PC1 contributing 60.76% and PC2 contributing 18.29%. Using PCA, the rice genotypes were grouped into four distinct clusters (Figure 9). The first group comprised the ASD7, Rathu Heenati, and PTB33 genotypes/lines, suggesting similarities in their characteristics concerning honeydew excretion, salivary sheaths, EPG analysis for WBPH feeding behavior, development, and survival rate, and trichome factors. The second group comprised the RD49 and UBN03078-101-342-148 genotypes/lines. The third group consisted of the TN1 and KDML105 genotypes, and the last group included the Mudgo, Babawee, and UBN03078-80-354-7 genotypes/lines. The results demonstrated that the distances between (resistant) ASD7 genotypes/lines were greater than those between UBN03078-80-354-7 genotypes/lines (susceptible). Thus, two varieties were used in the plant hormone and secondary metabolite profiling analysis.

### 2.6. Profiling of Plant Hormones and Secondary Metabolites

#### 2.6.1. Quantitative Changes in Phytohormone-Related Metabolites Following WBPH Infestation

Quantitative analysis indicated significant changes in the concentrations of gibberellin A3 (GA_3_), abscisic acid (ABA), and dihydrojasmonic acid (DHJA) in rice leaf samples following WBPH infestation, with distinct responses between the resistant genotype ASD7 and the susceptible genotype UBN03078-80-354-7 (Table 2). In the resistant genotype ASD7, GA_3_ content increased significantly following WBPH infestation, increasing from 14.00 ng mg^−1^ in control plants to 28.78 ng mg^−1^ in infested plants (*p* ≤ 0.01). A similar trend was observed in the susceptible genotype, where GA_3_ concentration increased significantly from 4.08 to 8.39 ng mg^−1^ following infestation (*p* ≤ 0.01), although the absolute levels remained substantially lower than those detected in ASD7. In contrast, the ABA content in ASD7 decreased significantly following WBPH infestation, declining from 0.310 to 0.107 ng mg^−1^ (*p* ≤ 0.01). Conversely, the susceptible genotype demonstrated a pronounced and significant increase in ABA concentration upon WBPH infestation, from 0.310 to 0.107 ng mg^−1^ (*p* ≤ 0.01), indicating a genotype-specific hormonal response to insect feeding. No significant changes in DHJA concentration were observed following WBPH infestation in either genotype.

#### 2.6.2. Changes in Secondary Metabolite Profiles in Rice Leaves Following WBPH Infestation

The accumulation of secondary metabolites in rice leaves differed markedly between the resistant genotype ASD7 and the susceptible genotype UBN03078-80-354-7 following infestation by the WBPH (Table 3). Genotype-dependent differences were observed in both the diversity and the magnitude of metabolite induction, as measured based on the ratio of WBPH-infested plants to uninfested controls. In the resistant genotype ASD7, WBPH infestation resulted in a pronounced and significant increase in multiple secondary metabolites. Notably, Imidazo [1,2-a]pyrazin-1-ium demonstrated the highest induction level, with a more than 10-fold increase compared with the control (*p* ≤ 0.01). Several fatty acid-related compounds, including octadec-9-enoic acid, γ-linolenic acid ethyl ester, palmitic acid, and palmitoleic acid, were also significantly elevated (ratios ranging from 1.84 to 2.32; *p* ≤ 0.01). In addition, defense-associated compounds such as ouabain, 2-hydroxybenzothiazole, and 3-nitro-1H-1,2,4-triazole-1,5-diamine were upregulated considerably in ASD7 following WBPH infestation (*p* ≤ 0.01). The phenylpropanoid-related compound trans-cinnamaldehyde was significantly, though moderately, induced (*p* ≤ 0.05). In contrast, the susceptible genotype UBN03078-80-354-7 exhibited minimal or negligible changes in secondary metabolite accumulation following WBPH infestation. Most compounds detected in ASD7 were absent or present at extremely low levels in the susceptible genotype, with WBPH/control ratios close to zero. Only trace induction was observed for γ-linolenic acid ethyl ester, 4-[3,5-di(tert-butyl)-1H-pyrazol-1-yl] benzoic acid, palmitoleic acid, ouabain, and 2-hydroxybenzothiazole. However, these changes were substantially lower than those observed in ASD7 and were not of strong statistical significance.

### 2.7. Gene Expression Analysis of SA/JA-Responsive Pathways Following WBPH Infestation

**Figure 10:**
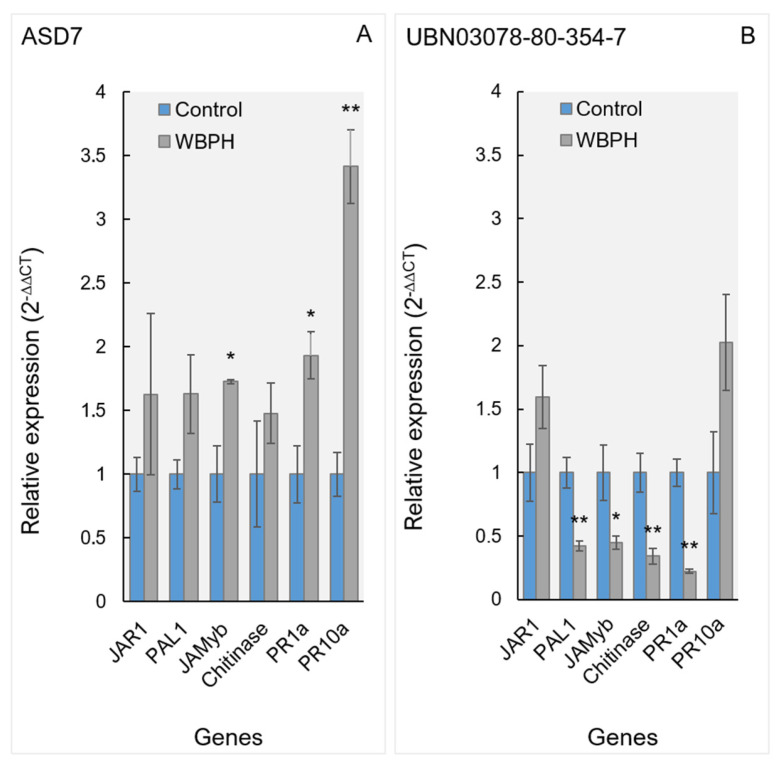
Relative expression of salicylic acid (SA)- and jasmonic acid (JA)-responsive genes in rice genotypes following infestation by the white-backed planthopper (*Sogatella furcifera*). (**A**) ASD7 (tolerant) and (**B**) UBN03078-80-354-7 (susceptible). Transcript levels of JAR1, PAL1, JAMyb, chitinase, PR1a, and *PR10a* were quantified via qRT-PCR in the youngest fully expanded leaves 14 days after WBPH infestation. Gene expression was normalized to OsActin and expressed relative to the uninfested control of the same genotype using the 2^−ΔΔCT^ method. Bars represent mean ± SE of three biological replicates (*n* = 3), each with three technical replicates. Asterisks indicate significant differences between infested and control plants within the same genotype (* *p* ≤ 0.05; ** *p* ≤ 0.01; Student’s *t*-test).

The expression patterns of salicylic acid (SA)- and jasmonic acid (JA)-responsive genes differed markedly between the tolerant genotype ASD7 and the susceptible genotype UBN03078-80-354-7 following infestation by the WBPH (Figure 10). In ASD7, WBPH infestation induced a significant upregulation of multiple defense-related genes compared with the uninfested control. Transcript levels of JAMyb and *PR1a* were significantly increased (*p* ≤ 0.05), whereas *PR10a* demonstrated a robust induction, reaching more than threefold higher expression relative to the control (*p* ≤ 0.01). Moderate but consistent increases were also observed for *JAR1*, *PAL1*, and *chitinase*, indicating coordinated activation of both JA- and SA-associated defense pathways in the tolerant genotype.

In contrast, the susceptible genotype UBN03078-80-354-7 demonstrated a markedly different transcriptional response to WBPH infestation. Expression levels of *PAL1*, *JAMyb*, chitinase, and *PR1a* were significantly downregulated in infested plants compared with their respective controls (*p* ≤ 0.05 or *p* ≤ 0.01). Although *PR10a* indicated a slight increase in expression following infestation, this induction was substantially lower than that observed in ASD7. No significant induction of *JAR1* was detected in the susceptible genotype.

## 3. Discussion

### 3.1. Honeydew Excretion and Salivary Sheath Formation

Honeydew excretion and salivary sheath formation are established indirect indicators of feeding intensity in sap-sucking insects. In this study, rice genotypes differed significantly in both parameters, indicating variation in host suitability to WBPH. PTB33 supported greater honeydew excretion and salivary sheath numbers, indicating efficient probing and sustained phloem ingestion. These observations align with the role of honeydew as a proxy for phloem acceptance and salivary sheaths as markers of successful stylet pathway construction [7,29,30]. In contrast, Rathu Heenati and Mudgo exhibited reduced honeydew output and fewer salivary sheaths, suggesting impaired probing and restricted phloem ingestion. Such reductions are commonly associated with physical and biochemical resistance traits that limit stylet penetration or reduce phloem suitability [31,32]. The positive correspondence between honeydew excretion and salivary sheath formation across genotypes further indicates that phloem ingestion success is contingent upon early probing events. Similar patterns have been reported in resistant rice challenged by WBPH and BPH, where reduced feeding correlates with impaired insect performance [33,34].

Honeydew excretion and salivary sheath formation assays revealed clear genotype-dependent differences in the feeding behavior of *Sogatella furcifera*, reflecting variation in host suitability among rice varieties (Figure 1A,B). Honeydew area is commonly used as an indirect indicator of sap ingestion, whereas the number of salivary sheaths reflects probing frequency and stylet pathway activities during host penetration [35]. However, these parameters primarily capture short-term insect responses during the host establishment phase rather than sustained feeding success.

PTB33 exhibited the largest honeydew drop area (Figure 1A), which might superficially suggest high susceptibility. In contrast, the same variety induced the highest number of salivary sheaths (Figure 1B), indicating frequent probing and repeated stylet insertions. Such feeding behavior is typically associated with difficulty in locating or maintaining access to phloem tissues, rather than effective phloem feeding [3]. Moreover, honeydew excretion does not exclusively represent phloem sap ingestion, as xylem-derived excretions may increase under conditions of feeding disruption or osmotic stress [35]. Therefore, the large honeydew area observed for PTB33 likely reflects compensatory non-phloem feeding rather than true host susceptibility.

### 3.2. Feeding Behavior by Electrical Penetration Graph

In the present study, apparent genotype-dependent differences in waveform occurrence and duration demonstrate that rice genotypes modulate WBPH feeding behavior at multiple stages, from stylet insertion and pathway activities to phloem and xylem ingestion. The identification of six distinct waveform patterns (Np, N1–N2, N3, N4-a, N4-b, and N5) aligns with previous characterizations of planthopper feeding behavior and confirms the reliability of waveform interpretation in this system. Variation in Np events among genotypes suggests differences in host acceptance and in surface-level cues that influence initial feeding decisions. Prolonged Np durations in specific genotypes indicate prolonged searching or hesitation before stylet insertion, typically associated with antixenosis-based resistance mediated by surface wax composition, trichome density, or deterrent metabolites [7,29,36,37].

Significant differences in the frequency and duration of pathway-phase waveforms (N1–N2 and N3) highlight genotype-specific effects on stylet penetration through epidermal and mesophyll tissues. Genotypes showing higher frequencies or extended durations of these waveforms likely impose greater mechanical or biochemical constraints on stylet progression. Such constraints have been linked to reinforced cell walls, increased silica deposition, and altered lignin or phenolic profiles in resistant rice genotypes, which collectively impede stylet movement and increase the metabolic costs for planthoppers [27,31]. Prolonged N3 durations in some genotypes further suggest repeated stylet adjustments or unsuccessful attempts to locate vascular tissues, a hallmark of pre-phloem resistance. Phloem-related waveforms (N4-a and N4-b) provided critical insights into post-phloem resistance mechanisms. Genotype-dependent variation in both the frequency and duration indicates differential phloem acceptance and sap ingestion efficiency. Prolonged N4-a durations are generally associated with increased salivation into sieve elements, often reflecting insect attempts to overcome phloem-based defenses such as callose deposition or phloem occlusion. In contrast, reduced frequency or duration of N4-b events in resistant genotypes suggests impaired sustained phloem sap ingestion, consistent with antibiosis mechanisms that restrict nutrient acquisition and reduce insect fitness [36,37,38,39]. These findings align with recent reports demonstrating that resistance genes and quantitative resistance loci in rice frequently target phloem-level interactions rather than causing the immediate cessation of feeding.

Collectively, the combined analysis of waveform number (NEWI), duration per event (WDEI), and cumulative duration per insect (WDI) reveals that resistance to WBPH is expressed through multiple interacting mechanisms operating across distinct feeding stages. Resistant genotypes often disrupt feeding early by prolonging NP and pathway phases and further restrict insect performance by reducing sustained phloem sap ingestion [36,37]. Such multi-layered resistance is highly advantageous for durable pest management, as it exerts continuous but moderate pressure on insect populations and may reduce the likelihood of rapid resistance breakdown.

### 3.3. Survival and Development Bioassays in WBPH

Survival and developmental traits provide integrative measures of host suitability for sap-feeders. In this study, WBPH survival and development differed significantly among rice genotypes, indicating genotype-dependent antibiosis. Early instars demonstrated negligible mortality across all genotypes, consistent with reports that initial nymphal stages rely on maternal reserves and exhibit limited feeding activity [7,38]. Divergent survival trajectories from the third instar onward suggest that resistance factors become effective as feeding intensity increases.

Pronounced reductions in survival on ASD7, Babawee, and Rathu Heenati support antibiosis-based resistance, a pattern frequently associated with phloem-mediated defensive traits that restrict nutrient acquisition [27,39]. In contrast, high survival and rapid development on TN1 and KDML105 indicate high host suitability and the absence of antibiosis [30]. Several genotypes, including RD49 and UBN03078-80-354-7, exhibited intermediate survival, suggesting partial resistance, underscoring their value in breeding programs [40]. Prolonged nymphal development and reduced adult longevity on resistant genotypes indicate nutritional stress or chronic exposure to plant defenses, highlighting trans-stadial effects of antibiosis [7,31]. Overall, the survival and developmental assays demonstrate mid- to late-instar antibiosis as a key resistance mechanism, complemented by feeding-based metrics from honeydew and EPG analyses.

### 3.4. The Biological Rice Genotypes Factor 

Scanning electron microscopy revealed substantial variation in trichome density among rice genotypes, particularly on the adaxial leaf sheath, which is the primary feeding site of WBPH. Genotypes such as TN1 and PTB33 exhibited significantly higher macrotrichome and total trichome densities, whereas resistant genotypes, including ASD7, Mudgo, and Rathu Heenati, showed consistently lower trichome densities. These findings suggest that trichomes alone do not confer direct resistance to WBPH; rather, they modulate insect settling and probing behavior in a genotype-dependent manner. Trichomes are considered to represent a first line of defense by acting as physical barriers that interfere with insect attachment, movement, and stylet insertion, thereby contributing to antixenosis [41,42]. However, high trichome density does not necessarily confer resistance. In some host–herbivore systems, dense trichomes may even facilitate insect settling by providing shelter or anchorage points, particularly for small-bodied sap-feeders [42]. This dual role aligns with the observed susceptibility of TN1 despite its high trichome density, indicating that physical defenses must operate in conjunction with chemical and physiological traits to effectively restrict WBPH feeding.

### 3.5. The Correlation and Principal Component Analysis Between Factors

Correlations underscore the importance of integrating physical and behavioral metrics when evaluating resistance. Previous studies have demonstrated that surface traits, including trichome density and cuticular composition, can modulate the frequency and duration of EPG waveforms associated with pathway and phloem phases, thereby influencing overall feeding success [36,37,38,39]. Nevertheless, the present results suggest that trichomes alone are insufficient to prevent sustained phloem ingestion, reinforcing the need for post-penetration defenses. The honeydew excretion and salivary sheath assays provided rapid and synergistic insights into WBPH feeding performance and host plant suitability. These methods effectively differentiated resistant, moderately resistant, and susceptible genotypes, supporting their utility as reliable phenotyping tools in rice resistance breeding programs. When integrated with advanced techniques such as EPG analysis and biochemical profiling, these assays contribute to a robust, multi-level framework for elucidating resistance mechanisms against WBPH and improving sustainable rice pest management strategies.

### 3.6. Plant Hormone and Secondary Metabolite Profiling

Quantitative phytohormone analysis revealed contrasting hormonal responses between the resistant genotype ASD7 and the susceptible genotype UBN03078-80-354-7 following WBPH infestation. The significant increase in GA_3_ in both genotypes indicates that WBPH feeding modulates growth-related signaling pathways. However, the substantially higher GA_3_ levels in ASD7 may reflect a compensatory growth response that offsets feeding damage, contributing to tolerance rather than direct resistance [43]. In contrast, ABA exhibited opposing trends between genotypes. The reduction in ABA in ASD7 following infestation aligns with enhanced defense activation, as ABA antagonizes JA and SA-mediated defense pathways. Conversely, the pronounced ABA accumulation in the susceptible genotype suggests the suppression of effective defense signaling, thereby facilitating WBPH feeding and performance. Such antagonistic interactions between the ABA and JA/SA signaling pathways are well established in plant–insect interactions and are considered a key determinant of resistance outcomes [44,45].

Secondary metabolite profiling further revealed pronounced genotype-dependent differences in chemical defenses. While WBPH infestation triggered substantial accumulation of multiple defense-associated metabolites in ASD7, the susceptible genotype indicated minimal or negligible induction. The robust upregulation of fatty acid-related compounds, phenylpropanoids, and heterocyclic defense molecules in ASD7 suggests the activation of multiple biosynthetic pathways that collectively contribute to antibiosis by reducing insect survival, delaying development, and restricting feeding efficiency.

Fatty acid derivatives and phenylpropanoid-related compounds are implicated in direct toxicity, feeding deterrence, and reinforcement of cell wall defenses against herbivores [31,46]. The dramatic induction of certain compounds, such as imidazo [1,2-a]pyrazin-1-ium and ouabain-like molecules, further supports the presence of potent chemical defenses in *ASD7*. In contrast, the minimal metabolic response in the susceptible genotype suggests a failure to mount effective inducible defenses upon WBPH attack.

### 3.7. Gene Expression Profiling of SA/JA Signaling Pathways 

Gene expression analysis corroborated the metabolomic and hormonal findings by demonstrating the coordinated activation of SA- and JA-responsive pathways in *ASD7*. The robust induction of *PR10a*, along with the upregulation of *JAMyb*, *PR1a*, *JAR1*, *PAL1*, and *chitinase*, indicates a broad-spectrum defense response that integrates both SA- and JA-mediated signaling. Such coordinated activation is a hallmark of durable resistance against piercing–sucking insects, which often evade or suppress single-pathway defenses [47,48]. In contrast, the downregulation of key defense genes in the susceptible genotype suggests active suppression or a failure of defense signaling, consistent with elevated ABA accumulation and insufficient induction of secondary metabolites. The nominal induction of *PR10a* alone appears insufficient to confer effective resistance, highlighting the importance of network-level defense activation rather than isolated gene responses.

## 4. Materials and Methods

The experiment was conducted in a laboratory and in a field at Suranaree University of Technology (14°58′14.38″ N 102°06′7.06″ E), Nakorn Ratchasima, Thailand.

### 4.1. Plant Materials and Insect Colony

Ten rice genotypes/lines were utilized for feeding and growth assays. Seeds of Rathu Heenati, RD49, Babawee, PTB33, Mudgo, ASD7, KDML105, TN1, UBN03078-80-354-7, and UBN03078-101-342-4-148 were obtained from the Ubon Ratchathani Rice Research Center and the Pathum Thani Rice Research Center in Thailand. A panel of rice genotypes was assembled, comprising the resistant donors (e.g., PTB33, Rathu Heenati) and breeding lines under evaluation, along with a susceptible check (e.g., TN1). Seeds were surface-sterilized with 1% sodium hypochlorite for 2 min, rinsed 3×, and germinated on moist paper for 48 h. Seedlings were subsequently transplanted into 10 cm pots (two plants per pot) filled with sterilized loam/peat/sand (2:1:1, *v*/*v*/*v*) and grown in a controlled greenhouse at 28 ± 2 °C, 60 ± 10% relative humidity (RH) and a 14:10 L:D photoperiod, with non-limiting nutrients provided weekly using half-strength Hoagland solution. Unless stated otherwise, plants were used at the tillering stage (25–30 days after sowing); panicle-stage assays were repeated at heading to test tissue/stage specificity.

White-backed planthoppers were obtained from the Ubon Ratchathani Rice Research Center. Colonies were maintained and multiplied on seedlings (≤14-day-old) of the susceptible genotypes TN1 and Khao Dawk Mali 105 (KDML105) under controlled conditions (28 °C, 60–70% RH, 14:10 light/dark photoperiod) in insect-proof cages. For bioassays, first-instar nymphs were used for growth and development tests, and fourth-instar nymphs were used for feeding rate and honeydew excretion assays. Unless stated otherwise, cohorts were synchronized via daily collections of newly molted nymphs.

### 4.2. Honeydew Excretion Assays

To quantify feeding intensity, honeydew excretion by WBPH was measured across 10 rice genotypes in a controlled experiment. A completely randomized design (CRD) was employed. Twenty biological replicates were prepared (one nymph per replicate). All assays were conducted at the Scientific and Technological Equipment Center, Suranaree University of Technology, at 28 °C and 60–70% RH. Fourth-instar nymphs were gently transferred to the leaf sheath of a test plant and confined using a ventilated clip cage. The exposure lasted 24 h (tillering stage). Honeydew was collected on Whatman No. 1 filter paper placed beneath the confined insect. After 24 h, the papers were immersed in a 0.1% (*w*/*v*) ninhydrin solution (ethanol solvent), air-dried at room temperature until purple spots developed (indicating amino-acid reaction), and then digitized using a flatbed scanner with fixed resolution and lighting [49]. Quantification involved measuring the honeydew area (mm^2^) from images using ImageJ version 1.54, National Institutes of Health, Bethesda, MD, USA. Calibration was performed with a stage micrometer, and the thresholding parameters were kept constant across samples. For each replicate, the total stained area per insect per 24 h was recorded as the response variable (Figure S2).

### 4.3. Leaf Staining Assay (Eosin) for Visualizing Feeding Punctures (Salivary Sheath Method)

To quantify planthopper stylet pathway traits and feeding success, salivary sheaths were enumerated in the leaf sheath tissue of the same genotype panel under controlled conditions. Plants at tillering (45 days after sowing) were used. Fourth-instar WBPH nymphs were starved for two hours (moistened air only) before assays to standardize motivation. A CRD was applied, with *n* = 20 biological replicates per genotype (one nymph per plant/leaf area). Assays were conducted at the Scientific and Technological Equipment Center, Suranaree University of Technology, at 28 °C and 60–70% RH. A ventilated clip cage (Ø 2.5–3.0 cm) was attached to the mid-leaf sheath (the primary feeding site). One fourth-instar nymph was confined for eight hours during the photophase. Following exposure, the insects were removed. Leaf sheath segments (≈2–3 cm) were excised and cleared in ethanol/acetic acid (3:1, *v*/*v*) for 30 min then rinsed twice in 70% ethanol and once in distilled water (1–2 min each). Leaves were immersed in 1% (*w*/*v*) eosin Y (in distilled water) for 10 min at room temperature, rinsed three times in distilled water (30 s each) to remove excess dye, and air-dried on lint-free paper (≈5 min). Pilot tests can be used to adjust staining to 8–12 min depending on cuticle thickness. Stained areas were examined under a stereomicroscope (e.g., 20–40×). Eosin-positive feeding punctures appeared as distinct red/crimson dots along the stylet pathway. For each replicate, we recorded the puncture count per field (fixed field of view), feeding puncture density (punctures cm^−2^; using a 1 cm^2^ graticule or calibrated image), and the proportion of fields with ≥1 puncture (host acceptance proxy). When needed, images were captured (with fixed illumination and magnification) and analyzed in ImageJ to confirm counts. Unstained leaves served as negative controls to verify the background. The response variable was feeding puncture density (cm^−2^) per replicate [50].

### 4.4. Electrical Penetration Graph Recordings of Feeding Behavior

This experiment aimed to quantify and compare the feeding behavior of the 10 rice genotypes/lines using DC-EPG. Feeding-behavior experiments were conducted using a Giga-8 DC electropenetrography (EPG) system (EPG Systems, Wageningen, The Netherlands) with an input resistance of 109 Ω (1 GΩ) and an adjustable plant voltage. The cassava plant and the insect with the EPG probe were placed within a Faraday cage (1.5 × 2 × 1.5 m^3^) to block electrical noise. The system was installed in a temperature-controlled room at 28 ± 2 °C and 60 ± 5% RH. EPG signals were recorded using Stylet+d software, with the signal range adjusted from −5 to +5 V, and displayed on a computer. Fourth-instar nymphs of WBPH were placed in a glass vial and immobilized on ice for 5–10 s before being connected to a gold wire electrode (2.5 cm long, 12 μm diameter) (EPG Systems, Wageningen, The Netherlands), with water-based silver glue (Wageningen University) applied to the insect’s pronotum. The other end of the wire was attached to a copper electrode (1 mm diameter, 2.5 cm long) (Sigmund Cohn Corp, Mt Vernon, NY, USA), which was connected to the EPG probe. The WBPH was placed on the abaxial leaf surface and fixed with parafilm. Signal adjustments were made if needed, and data were recorded using Stylet+d software. Each WBPH was observed for three hours daily, with 20 WBPH tested per genotype in a completely randomized design. Annotated waveforms (NP, stylet pathway, phloem salivation, phloem ingestion, intracellular puncture, and xylem ingestion) were analyzed using EPG Systems software and a modified Ebert 3.0 program in SAS Enterprise Guide 7.1, SAS 9.4 statistical software.

EPG data recorded using EPG Systems Stylet+d were manually annotated utilizing EPG Systems Stylet+a software. The annotated waveforms included Np, pathway in epidermis/mesophyll (N1, N2), phloem salivation or sieve-element salivation (N4-a), sustained phloem sap ingestion or phloem ingestion (N4-b), and xylem ingestion (N5). The waveform patterns were categorized based on amplitude, relative voltage level, R/emf origin, frequency, and waveform context, as described in previous EPG studies on brown planthoppers, planthoppers, the whitefly *B. tabaci*, and leafhoppers [37,38,39,41,42,43,44,45]. Annotation files were then directly transferred to a modified version of the Ebert 3.0 program in SAS Enterprise Guide 7.1, SAS 9.4 statistical software (SAS Institute, Cary, NC, USA), for further analysis, producing the same parameters as the popular Sarria Excel workbook [51,52].

### 4.5. Development and Survival Bioassays (First Instar to Adult)

This assay was designed to compare the nymphal development and survival of WBPH across 10 rice genotypes/lines. Each seedling was grown individually in a 7–10 cm pot (sterilized substrate), watered daily, and kept free of pesticides. Newly hatched first-instar (N1) nymphs of WBPH (≤24 h old) were utilized. Assays were conducted in a controlled-environment room at 28 °C and 75% RH, with a 12:12 light/dark photoperiod. A completely randomized design (CRD) was employed, with *n* = 20 biological replicates per genotype (one nymph per seedling = one replicate). A single N1 nymph was gently transferred with a camel-hair brush to the leaf sheath/collar of a seedling and confined using a ventilated clip cage (Ø 2.5–3.0 cm) or a mesh sleeve that allowed free movement on that plant only. Plants were spaced to prevent cage contact. Each replicate was inspected at the same time every 24 h. For each nymph, we recorded the instar transitions (N1→N2→N3→N4→N5→adult) based on exuviae and morphology; instar duration (days); cumulative development time to adult (days); stage-specific mortality (cause noted if evident: desiccation, escape, handling); adult emergence (%); and sex ratio [53,54]. Plants were replaced with fresh seedlings every 3–4 days or immediately if wilting occurred. Escaped or physically injured nymphs were recorded and excluded (censored) a priori (Figure S3).

### 4.6. Biophysical Factors 

The morphology of the abaxial surface of the ten rice leaves—RD49, Babawee, Rathu Heenati, PTB33, Mudgo, ASD7 (putative resistant), TN1, KDML105, UBN03078-80-354-7 (susceptible), and UBN03078-101-342-4-148 (susceptible)—was analyzed for trichome density. Samples from the leaf sheath at the stem of 45-day-old plants were fixed in 37% formaldehyde overnight, washed in phosphate-buffered saline, and dehydrated in an ethanol series (70%, 80%, 95%, and 100%). Samples were critical-point-dried and coated with a gold–palladium alloy. The fixed surfaces of the leaves were then observed under a cutting-edge scanning electron microscope (SEM) (SEM™, FEI, Quanta450, Netherlands). Trichome density was assessed manually at 0.3 mm^2^ intervals, with 10 biological replications and five images per leaf [50].

### 4.7. Pearson Correlation and Principal Component Analysis Between the Parameters

A correlation analysis (Pearson correlation) was conducted on the mean values for honeydew excretion, salivary sheath formation, EPG analysis for WBPH feeding behavior, development and survival rate, and trichome factors to identify the relationships among these parameters in this study. Differences were considered significant at the 5% level. PCA was used to evaluate the relationships among the 10 rice genotypes. Principal component analysis and cluster analysis were utilized as multivariate statistical methods to discern patterns among the investigated rice genotypes based on multiple parameters, including honeydew excretion, salivary sheath formation, EPG analysis for WBPH feeding behavior, development and survival rate, and trichome factors. Pearson correlation analysis, PCA, and cluster analysis were performed using OriginPro 2024 (OriginLab Corporation, Northampton, MA, USA).

Based on the results of correlation, PCA, and hierarchical cluster analyses, two rice genotypes exhibiting contrasting resistance phenotypes (resistant and susceptible) were selected for further studies. These selected genotypes were used to compare plant hormone and secondary metabolite profiles and analyze gene expression in SA- and JA-responsive pathways.

### 4.8. Plant Hormone and Secondary Metabolite Analysis

Leaves from the resistant rice genotypes ASD7 and the susceptible line UBN03078-80-354-7 were collected from each experimental set (*n* = 3 biological replicates per genotype). Samples were immediately frozen in liquid nitrogen, ground to a fine powder, and freeze-dried to a constant weight. Freeze-dried leaf powder (35 mg) was used for hormone extraction. Leaf powder was extracted with 3.5 mL methanol containing 0.01% (*v*/*v*) formic acid, vortexed for 30 s, and incubated at −20 °C for 48 h. Extracts were centrifuged at 14,000× *g* for 60 min at 4 °C, and supernatants were collected. To reduce matrix effects, extracts were diluted with acetonitrile and ultrapure water and subsequently purified using solid-phase extraction (SPE) cartridges pre-conditioned with methanol and water. Analytes were eluted with acetonitrile, evaporated to dryness under vacuum, reconstituted in methanol, diluted (1:1, *v*/*v*) with 0.2% formic acid in water, and filtered (0.22 µm PTFE) before analysis.

UHPLC–Orbitrap MS was used for phytohormone analysis, with a UHPLC system coupled to a Q-Exactive Orbitrap mass spectrometer (Thermo Fisher Scientific, Waltham, MA, USA) equipped with a C18 column (2.1 mm × 100 mm, 1.9 µm particle size). Mobile phases consisted of water (A) and acetonitrile (B), both containing 0.1% formic acid. A gradient elution was applied from 10% to 100% B at a flow rate of 0.3 mL min^−1^. The column temperature was maintained at 40 °C, the injection volume was 5 µL, and the autosampler temperature was set to 10 °C. The ion polarity was in positive mode, the capillary temperature was 350 °C, and the spray voltage was 3.8 kV. Data were acquired in Full-MS and targeted PRM modes as described in [55].

Calibration and quantification involved preparing stock solutions (1 mg mL^−1^) of GA, ABA, and DHJA in methanol and diluting them in series to generate external calibration standards (0.25–40 mg mL^−1^). Quantification was conducted using PRM with the following precursor ions: GA (*m*/*z* 347.14891), ABA (*m*/*z* 265.14344), and DHJA (*m*/*z* 213.14852). Analytes were quantified based on extracted ion chromatograms (±5 ppm) using linear regression, and concentrations were expressed as mg g^−1^ dry weight. Procedural blanks and spiked samples were included for quality control.

### 4.9. Gene Expression Analysis of SA/JA-Responsive Pathways

The youngest fully expanded leaves of ASD7 (resistant) and UBN03078-80-354-7 (susceptible) were collected 14 days after insect infestation (WBPH), alongside uninfested controls. Leaves were flash-frozen and stored at −80 °C. RNA was extracted using the Plant Total RNA Mini Kit (Favorgen Biotech Corp., Ping Tung, Taiwan) following the manufacturer’s protocol, including on-column DNase treatment. RNA quality was verified (A_260_/_280_ ≈ 2.0; intact rRNA bands), and 500 ng total RNA was reverse-transcribed with iScript Reverse Transcriptase Supermix (Bio-Rad Laboratories, Hercules, CA, USA) to synthesize cDNA (20 µL reaction). SA/JA pathway markers were as follows: JAR1 (conjugates JA→JA-Ile), PAL1 (phenylpropanoid entry enzyme), JAMyb (JA/SA-responsive TF), chitinase (defense enzyme), *PR1a*, and *PR10a* (pathogenesis-related) (Table S1). OsActin served as the reference gene [56]. These genes were selected to represent a functional continuum from the essential activation of bioactive JA-Ile (JAR1) and transcriptional regulation (JAMyb) to the structural reinforcement of cell walls via the phenylpropanoid pathway (PAL1) and, finally, the production of direct defensive effectors (Chitinase, *PR1a*, *PR10a*) that target the physiological integrity of the herbivore [57,58,59,60,61,62,63,64,65].

Reactions (10–20 µL) contained SYBR Green master mix, gene-specific primers (Table S1), and 2 µL diluted cDNA. Cycling was performed using a real-time PCR system: 95 °C 3 min; 40 cycles of 95 °C 10 s/58–60 °C 20–30 s/72 °C 20–30 s. Melt-curve analysis was employed to verify specificity. No-RT and no-template controls were included. There were three biological replicates per genotype × infestation (*n* = 3). CT values were processed after baseline/threshold inspection; outliers due to melt-curve anomalies were excluded a priori. Relative expression was calculated as 2^−ΔΔCT^, normalized to OsActin and then to the uninfested control of the same genotype.

### 4.10. Statistical Analysis

For all experiments, the honeydew area, salivary sheath, mean development time (days) from N1 to adult, stage-specific mortality (%) per instar, adult emergence (%), and trichome density were analyzed utilizing one-way ANOVA with genotype as the factor (CRD). When significant, means were separated using Tukey’s honestly significant difference (HSD) test (*p* = 0.05) in SAS. If required, data were checked for normality and homoscedasticity; the Cox transformation was applied when assumptions were violated.

EPG waveforms were characterized based on feeding-behavior events as follows: Np, pathway in epidermis/mesophyll (N1, N2), phloem salivation or sieve-element salivation (N4-a), sustained phloem sap ingestion or phloem ingestion (N4-b), and xylem ingestion (N5). The variables of total probing duration (TPD), total waveform duration (TWD), frequency of waveform events per insect (NWEI), WDEI, and waveform duration per insect (WDI) were also calculated (mean ± standard error), as described in previous studies [66]. The variables were compared using the Tukey–Kramer test at *p* < 0.05. All analyses were performed using SAS V9.4. Pearson correlation analysis was conducted for the mean values of honeydew area, salivary sheath method, mean development time (days) from N1 to adult, stage-specific mortality (%) per instar, adult emergence (%), and trichome density using OriginPro 2024 software, with significance set at *p* < 0.05 [67].

## 5. Conclusions

This study demonstrates that rice resistance to *S. furcifera* is governed by the integrated action of physical and chemical defense mechanisms. Leaf surface traits contributed to antixenosis by modulating insect settling and probing behavior. In contrast, inducible hormonal signaling, secondary metabolite accumulation, and defense-related gene activation played a primary role in antibiosis by restricting feeding efficiency, survival, and development. Behavioral assays (honeydew excretion, salivary sheath formation, and EPG) aligned with life-history outcomes, validating their utility for resistance screening. Notably, gene expression analysis revealed robust upregulation of *PR10a* in resistant rice, highlighting its potential role as a molecular marker of WBPH resistance. Rice genotypes that exhibit coordinated antixenosis and antibiosis responses, such as *ASD7*, represent valuable sources of durable resistance. Overall, multi-trait phenotyping provides a robust framework for screening and deploying rice genotypes with sustainable resistance to WBPH.

## Figures and Tables

**Table 1:** Growth and developmental parameters of the white-backed planthopper (*Sogatella furcifera*) reared on 10 rice genotypes/lines: Rathu Heenati, RD49, Babawee, PTB33, Mudgo, ASD7, Khao Dawk Mali 105 (KDML105), TN1, UBN03078-80-354-7, and UBN03078-101-342-4-148.

Rice Varieties	Mean ± SEM (Days)						Sex Ratio (Male/Female)
	First-Instar Nymph	Second-Instar Nymph	Third-Instar Nymph	Fourth-Instar Nymph	Fifth-Instar Nymph	Adult	
KDML105	1.20 ± 0.36 d	1.19 ± 0.51	2.19 ± 1.12 bc	2.33 ± 0.47	6.43 ± 4.87	8.86 ± 2.71 ab	2:1
TN1	1.50 ± 0.49 d	1.20 ± 0.41	1.60 ± 0.83 c	2.13 ± 2.13	3.93 ± 3.90	7.4 ± 1.06 ab	2:1
UBN03078-80-354-7	2.24 ± 0.54 a	1.57 ± 0.51	1.05 ± 0.59 dc	2.86 ± 1.39	4.71 ± 3.78	4.71 ± 5.19 b	1:0.3
UBN03078-101-342-148	1.20 ± 0.41 d	1.24 ± 0.54	2.9 ± 2.55 ab	2.48 ± 2.27	3.67 ± 4.02	1.29 ± 0.04 c	1:1
PTB33	1.85 ± 0.59 b	1.65 ± 0.81	3.30 ± 1.38 a	3.60 ± 3.23	5.55 ± 4.60	2.15 ± 1.29 bc	1:1
RD49	1.40 ± 0.52 c	1.50 ± 0.53	2.20 ± 0.79 bc	1.2 ± 1.04	1.60 ± 1.73	NA	NA
Babawee	2.29 ± 0.46 a	1.95 ± 0.28	2.52 ± 0.81 ab	2.19 ± 1.20	6.24 ± 3.26	9.24 ± 0.83 ab	1:1.35
ASD7	1.50 ± 0.51 c	1.55 ± 0.86	2.55 ± 1.32 ab	2.18 ± 1.56	3.77 ± 1.68	4.64 ± 0.29 b	1:1.42
Mudgo	2.08 ± 0.28 ab	1.90 ± 0.20	1.46 ± 0.66 c	2.88 ± 1.08	4.54 ± 1.47	12.83 ± 4.29 a	1.1:1
Rathu Heenati	1.23 ± 0.31 d	1.83 ± 0.37	3.25 ± 1.47 a	2.50 ± 1.15	3.38 ± 1.35	5.54 ± 4.12 b	1:1.24
*p*-value	**	NS	**	NS	NS	**	

Values followed by different letters indicate significant differences at *p* ≤ 0.05 according to Duncan’s multiple range test (DMRT). ** indicates a statistically significant difference at *p* ≤ 0.05 according to Duncan’s multiple range test. NS in a table indicates a statistically significant difference between groups, where the *p*-value < 0.05.

**Table 2:** Quantitative analysis of the metabolites gibberellin A3 (GA_3_), abscisic acid (ABA), and dihydrojasmonic acid in rice leaf samples from all three treatments.

Metabolites and Treatments		Hormone Content (ng/mg^−1^)	
		ASD7	UBN03078-80-354-7(s)
Gibberellin A3 (GA3)	Control	13.997 ± 0.007	4.082 ± 0.002
	WBPH	28.780 ± 0.026 **	8.394 ± 0.008 **
Abscisic Acid (ABA)	Control	0.310 ± 0.003	0.313 ± 0.001
	WBPH	0.107 ± 0.002 **	0.907 ± 0.001 **
Dihydrojasmonic Acid	Control	0.0433 ± 0.003	0.009 ± 0.001
	WBPH	0.0432 ± 0.004	0.011 ± 0.000

SEM denotes standard error of the mean, and hormone content represents the calculated amount derived from the standard curve. *n* = 3. ** a statistically significant difference compared with the control (*p* ≤ 0.01), ** a highly significant statistical difference compared with the control (*p* ≤ 0.01), as determined using the *t*-test.

**Table 3:** Analysis of secondary metabolites in leaf samples of the resistant rice genotype ASD7 and the susceptible genotype UBN03078-80-354-7.

Compound	ASD7	UBN03078-80-354-7(s)
	(WBPH)/(Control) Ratio	(WBPH)/(Control) Ratio
Imidazo [1,2-a]pyrazin-1-ium	10.924 **	N/A
Octadec-9-enoic acid	2.167 **	N/A
γ-Linolenic acid ethyl ester	2.322 **	0.002
Palmitic acid	2.277 **	N/A
4-[3,5-di(tert-butyl)-1H-pyrazol-1-yl] benzoic acid	1.657 **	0.652
Palmitoleic acid	1.84 **	0.008
Ouabain	1.76 **	0.005
3-Nitro-1H-1,2,4-triazole-1,5-diamine	1.055 **	N/A
2-Hydroxybenzothiazole	1.264 **	0.006
Trans-Cinnamaldehyde	1.15 *	0.003

* A statistically significant difference compared with the control (*p* ≤ 0.05); ** a highly statistically significant difference compared with the control (*p* ≤ 0.01), as determined using the *t*-test.

## Data Availability

The original contributions presented in this study are included in the article/Supplementary Materials. Further inquiries can be directed to the corresponding author.

## Supplementary Materials

The following supporting information can be downloaded at: https://www.mdpi.com/article/10.3390/plants15050811/s1, Figure S1. The size of the honeydew area and the intensity of the honeydew color correspond to the white-backed planthopper (Sogatella furcifera) feeding activity. Figure S2. (A) Collection of white-backed planthopper (Sogatella furcifera) honeydew on Whatman filter paper that was placed at the bottom of insect cages, and (B) representative staining of honeydew spots on filter paper with 0.1% ninhydrin. Figure S3. The Rice phenology used for development and survival bioassays. Each seedling was grown individually in a 7–10 cm pot (sterilized substrate), watered daily, and kept free of pesticides. Table S1. Sequencing primer and the function of the genes.

## Abbreviations

The following abbreviations are used in this manuscript:

CRD	Completely Randomized Design
EPG	Electrical Penetration Graph
IRRI	International Rice Research Institution
JA	Jasmonic acid
RGSV	Rice grassy stunt virus
SEM	Scanning Electron Microscope
SA	Salicylic acid
SAS	Statistical Analysis System
v/v	Volume/volume percentage

## References

[1] Ye Y., Xiong S., Guan X., Tang T., Zhu Z., Zhu X., Hu J., Wu J., Zhang S. (2024). Insight into rice resistance to the brown planthopper: Gene cloning, functional analysis, and breeding applications. Int. J. Mol. Sci..

[2] Heong K.L., Hardy B. (2009). Planthoppers: New Threats to the Sustainability of Intensive Rice Production Systems in Asia.

[3] Sogawa K. (1982). The rice brown planthopper: Feeding physiology and host plant interactions. Annu. Rev. Entomol..

[4] Cheng J.A. (2009). Rice planthopper problems and relevant causes in China. Planthoppers: New Threats to the Sustainability of Intensive rice production systems in Asia.

[5] Matsumura M., Takeuchi H., Satoh M., Sanada-Morimura S., Otuka A., Watanabe T., Van Thanh D. (2009). Current status of insecticide resistance in rice planthoppers in Asia. Pest Manag. Sci..

[6] Bottrell D.G., Schoenly K.G. (2012). Resurrecting the ghost of green revolutions past: The brown planthopper as a recurring threat to high-yielding rice production in tropical Asia. J. Asia-Pac. Entomol..

[7] Horgan F.G., Crisol E. (2013). Hybrid rice and insect herbivores in Asia. Entomol. Exp. Appl..

[8] Painter R.H. (1951). Insect Resistance in Crop Plants.

[9] Smith C.M. (2005). Plant Resistance to Arthropods: Molecular and Conventional Approaches.

[10] Velusamy R., Heinrichs E.A., Medrano F.G. (1986). Greenhouse techniques to identify field resistance to brown planthopper. Nilaparvata lugens. Crop Sci..

[11] Cao T.T., Lu J., Lou Y.G., Cheng J.A. (2013). feeding-induced interactions between two rice planthoppers, *Nilaparvata lugens* and *Sogatella furcifera* (Hemiptera: Delphacidae): Effects on feeding and honeydew excretion. Environ. Entomol..

[12] Carrasco D., Larsson M.C., Anderson P. (2015). Insect host plant selection in complex environments. Curr. Opin. Insect Sci..

[13] Panda N., Khush G.S. (1995). Host Plant Resistance to Insects.

[14] Yoshihara T., Sogawa K., Pathak M.D., Juliano B.O. (1979). Soluble silicic acid as a sucking inhibitory substance in rice against the brown planthopper. Entomol. Exp. Appl..

[15] Pathak P.K., Saxena R.C. (1981). Honeydew excretion by Nilaparvata lugens as an indicator of feeding activity. Entomol. Exp. Appl..

[16] Mclean D., Kinsey M. (1964). A Technique for electronically recording aphid feeding and salivation. Nature.

[17] TjallingiI W.F. (1978). Mechanoreceptors of the aphid labium. Entomol. Exp. Appl..

[18] Tjallingii W.F. (1988). Electrical recording of stylet penetration activities. Aphids: Their Biology, Natural Enemies and Control.

[19] Seo B.Y., Kwon Y.H., Jung J.K., Kim G.H. (2009). Feeding behavior of the brown planthopper on resistant and susceptible rice varieties. Entomol. Exp. Appl..

[20] Seo B.Y., Jung J.K., Choi B.R., Park H.M., Lee S.W., Lee B.H. (2010). Survival rate and stylet penetration behavior of current Korean populations of the brown planthopper, *Nilaparvata lugens*, on resistant rice varieties. J. Asia Pac. Entomol..

[21] Lei W., Li P., Han Y., Gong S., Yang L., Hou M. (2016). EPG recordings reveal differential feeding behaviors in Sogatella furcifera in response to plant virus infection and transmission success. Sci. Rep..

[22] Chandrasekar K., Suresh S., Soundararajan R.P., Boopathi T. (2017). Feeding behavior of whitebacked planthopper, *Sogatella furcifera* (Horvath) on selected rice genotypes. J. Entomol. Zool. Stud..

[23] Kang Y., Koo H.N., Kim H.K., Kim G.H. (2022). Analysis of the feeding behavior and life table of *Nilaparvata lugens* and *Sogatella furcifera* (Hemiptera: Delphacidae) under sublethal concentrations of imidacloprid and sulfoxaflor. Insects.

[24] Heinrichs E.A., Medrano F.G., Rapusas H.R. (1985). Genetic Evaluation for Insect Resistance in Rice.

[25] War A.R., Paulraj M.G., Ahmad T., Buhroo A.A., Hussain B., Ignacimuthu S., Sharma H.C. (2012). Mechanisms of plant defense against insect herbivores. Plant Signal. Behav..

[26] Mitsuhara I., Matsufuru H., Ohshima M., Kaku H., Nakajima Y., Murai N., Natori S., Ohashi Y. (2008). Induced expression of PR10 genes in rice plants. Plant Mol. Biol..

[27] Du B., Zhang W., Liu B., Hu J., Wei Z., Shi Z., He R., Zhu L., Chen R., Han B. (2009). Identification and characterization of Bph14, a gene conferring resistance to brown planthopper in rice. Proc. Natl. Acad. Sci. USA.

[28] He Y., Chen L., Chen J., Zhang J., Chen L., Shen J., Zhu Y.C. (2011). Electrical penetration graph evidence thatpymetrozine toxicity to the rice brown planthopper is byinhibition of phloem feeding. Pest Manag. Sci..

[29] Backus E.A., Cline A.R., Ellerseick M.R., Serrano M.S. (2015). Lygus hesperus feeding behavior: New EPG waveforms and expanded behavioral meanings. Ann. Entomol. Soc. Am..

[30] Ning S., Yang L., Chen Z., Zhou H., Wang X., Li Z. (2022). Integrating EPG and honeydew analysis reveals resistance mechanisms in rice against planthoppers. J. Econ. Entomol..

[31] Li R., Weldegergis B.T., Li J., Jung C., Qu J., Sun Y., Qian H., Tee C., van Loon J.J.A., Dicke M. (2019). Virulence factors of insect herbivores and plant defense responses. New Phytol..

[32] Hu L., Ye M., Li R., Lou Y. (2023). Plant-mediated interactions between herbivores and rice defense signaling. Front. Plant Sci..

[33] Heinrichs E.A. (1986). Perspectives and directions for the continued development of insect-resistant rice varieties. Agric. Ecosyst. Environ..

[34] Qiu Y., Guo J., Jing S., Tang M., He G. (2011). Identification of antibiosis and antixenosis in rice varieties resistant to the brown planthopper. J. Econ. Entomol..

[35] Heong K.L., Sogawa K. (1989). Relationship between feeding activity and honeydew excretion of the brown planthopper *Nilaparvata lugens* (Stål) on rice plants. Appl. Entomol. Zool..

[36] Roddee J., Wangkeeree J., Hanboonsong Y. (2024). Identification and Evaluation of Sugarcane Cultivars for Antixenosis Resistance to the Leafhopper *Yamatotettix flavovittatus* Matsumura (Hemiptera: Cicadellidae). Plants.

[37] Pimkornburee S., Pombud S., Buensanteai K., Namanusart W., Aiamla-or S., Roddee J. (2024). Impact of cassava cultivars on stylet penetration behavior and settling of *Bemisia tabaci* Gennadius (Hemiptera: Aleyrodidae). Plants.

[38] Wu Y., Zhang Y., Xu J., Li Z., Wang X., He G. (2021). Behavioral and physiological mechanisms of brown planthopper resistance in rice. Pest Manag. Sci..

[39] Zhang C., Luo S., Xu J., Lou Y., He G. (2023). Advances in understanding rice resistance mechanisms to planthoppers. Plants.

[40] Crowder D.W., Jabbour R. (2014). Relationships between biodiversity and biological control in agroecosystems. Curr. Opin. Insect Sci..

[41] Levin D.A. (1973). The role of trichomes in plant defense. Q. Rev. Biol..

[42] Karabourniotis G., Liakopoulos G., Nikolopoulos D., Bresta P. (2020). Protective and defensive roles of non-glandular trichomes against multiple stresses: Structure–function coordination. J. Plant Physiol..

[43] Huot B., Yao J., Montgomery B.L., He S.Y. (2014). Growth–defense tradeoffs in plants: A balancing act to optimize fitness. Mol. Plant.

[44] Robert-Seilaniantz A., Grant M., Jones J.D.G. (2011). Hormone crosstalk in defense. Annu. Rev. Phytopathol..

[45] De Vleesschauwer D., Xu J., Höfte M. (2014). Hormone-mediated defense networking in rice. Front. Plant Sci..

[46] Erb M., Reymond P. (2019). Plant–insect molecular interactions. Annu. Rev. Plant Biol..

[47] Zhou G., Qi J., Ren N., Cheng J., Erb M., Mao B., Lou Y. (2019). Silencing insect herbivore genes by plant-mediated RNAi: A new dimension of plant defense. Plant Cell.

[48] Fujita D., Kohli A., Horgan F.G. (2013). Rice resistance to planthoppers and leafhoppers. Crit. Rev. Plant Sci..

[49] Xiao C., Hu J., Ao Y.T., Cheng M.X., Gao G.J., Zhang Q.L., He G.C., He Y.Q. (2016). Development and evaluation of near-isogenic lines for brown planthopper resistance in rice cv. 9311. Sci. Rep..

[50] Roddee J., Wangkeeree J., Backus E.A., Hanboonsong Y. (2023). Probing behavior of the leafhopper analyzed through DC electropenetrography and microscopy. J. Insect Physiol..

[51] Sarria E., Cid M., Garzo E., Fereres A. (2009). Excel workbook for automatic parameter calculation of EPG data. Comput. Electron. Agric..

[52] Ebert T.A., Backus E.A., Cid M., Fereres A., Rogers M.E. (2015). A new SAS program for behavioral analysis of electrical penetration graph data. Comput. Electron. Agric..

[53] Horgan F.G., Amin I., Cristóbal M.S., Carvalho L.M., Barrion A.T. (2018). Resistance and tolerance to the brown planthopper Nilaparvata lugens (Hemiptera: Delphacidae) in rice: Estimating functional relationships among plant resistance traits. Crop Prot..

[54] Zhang J.-H., Liu Y., Wang L. (2020). Evaluation of rice lines with Bph resistance genes against Nilaparvata lugens: Effects on nymph survival and developmental duration. Agriculture.

[55] Yingchutrakul Y., Sittisaree W., Mahatnirunkul T., Chomtong T., Tulyananda T., Krobthong S. (2021). Cosmeceutical potentials of *Grammatophyllum speciosum* extracts: Anti-inflammations and anti-collagenase activities with phytochemical profile analysis using an untargeted metabolomics approach. Cosmetics.

[56] Li C., Ni P., Francki M., Hunter A., Zhang Y., Schibeci D., Li H., Tarr A., Wang J., Cakir M. (2004). Genes controlling seed dormancy and pre-harvest sprouting in a rice-wheat-barley comparison. Funct. Integr. Genom..

[57] van Loon L.C., Rep M., Pieterse C.M.J. (2006). Significance of inducible defense-related proteins in infected plants. Annu. Rev. Phytopathol..

[58] Fu Z.Q., Dong X. (2013). Systemic acquired resistance: Turning local infection into global defense. Annu. Rev. Plant Biol..

[59] Xie Y., Chen Z., Brown R.L., Bhatnagar D. (2014). Expression and functional analysis of rice PR10 genes in defense responses. Plant Mol. Biol..

[60] Wang Y., Li J., Li Y., Zhu S., Zhang Y. (2020). PR10 proteins in plant defense: Structure, function and regulation. Plants.

[61] Wasternack C., Song S. (2017). Jasmonates. Biosynthesis, metabolism, and signaling by proteins activating and repressing transcription. J. Exp. Bot..

[62] Niu Y., Figueroa P., Browse J. (2011). Characterization of JAZ-Interacting MYC transcription factors in jasmonate signaling. Plant Cell.

[63] Li C., Wang Y., Liu L., Hu Y., Zhang F., Mergen S., Wang G. (2021). The OsMYB transcription factor family in rice: Functional roles in hormone signaling and stress responses. Plants.

[64] Sharma H.C., Sujana G., Rao D.M. (2018). Morphological and biochemical components of resistance to insect pests in plants. Agric. Res..

[65] Grover A. (2012). Plant chitinases: Genetic diversity and physiological roles. Crit. Rev. Plant Sci..

[66] Backus E.A., Cline A.R., Ellerseick M.R., Serrano M.S. (2007). Lygus hesperus (Hemiptera: Miridae) feeding on cotton: New methods and parameters for analysis of nonsequential electrical penetration graph data. Ann. Entomol. Soc. Am..

[67] Moberly J.G., Bernards M.T., Waynant K.V. (2018). Key features and updates for Origin 2018. J. Cheminform..

