# Supplementary material for: Screening and Evaluation of Rice to Assess Antibiosis and Antixenosis Resistance to White-Backed Planthopper (Sogatella furcifera)
Source: Plants (Basel). 2026 Mar 6;15(5):811. doi: 10.3390/plants15050811 (PMC12986885; doi:10.3390/plants15050811)
Supplement: Supplementary file 1 [file plants-15-00811-s001.zip › plants-4138119-supplementary.pdf]

Supplementals

Screening and Evaluation of Rice to Assess Antibiosis and Antixenosis Resistance to White-Backed Planthopper (*Sogatella furcifera*)

Supplements Results

Supplemental Figure

Figure S1. The size of the honeydew area and the intensity of the honeydew color correspond to the white-backed planthopper (*Sogatella furcifera*) feeding activity.

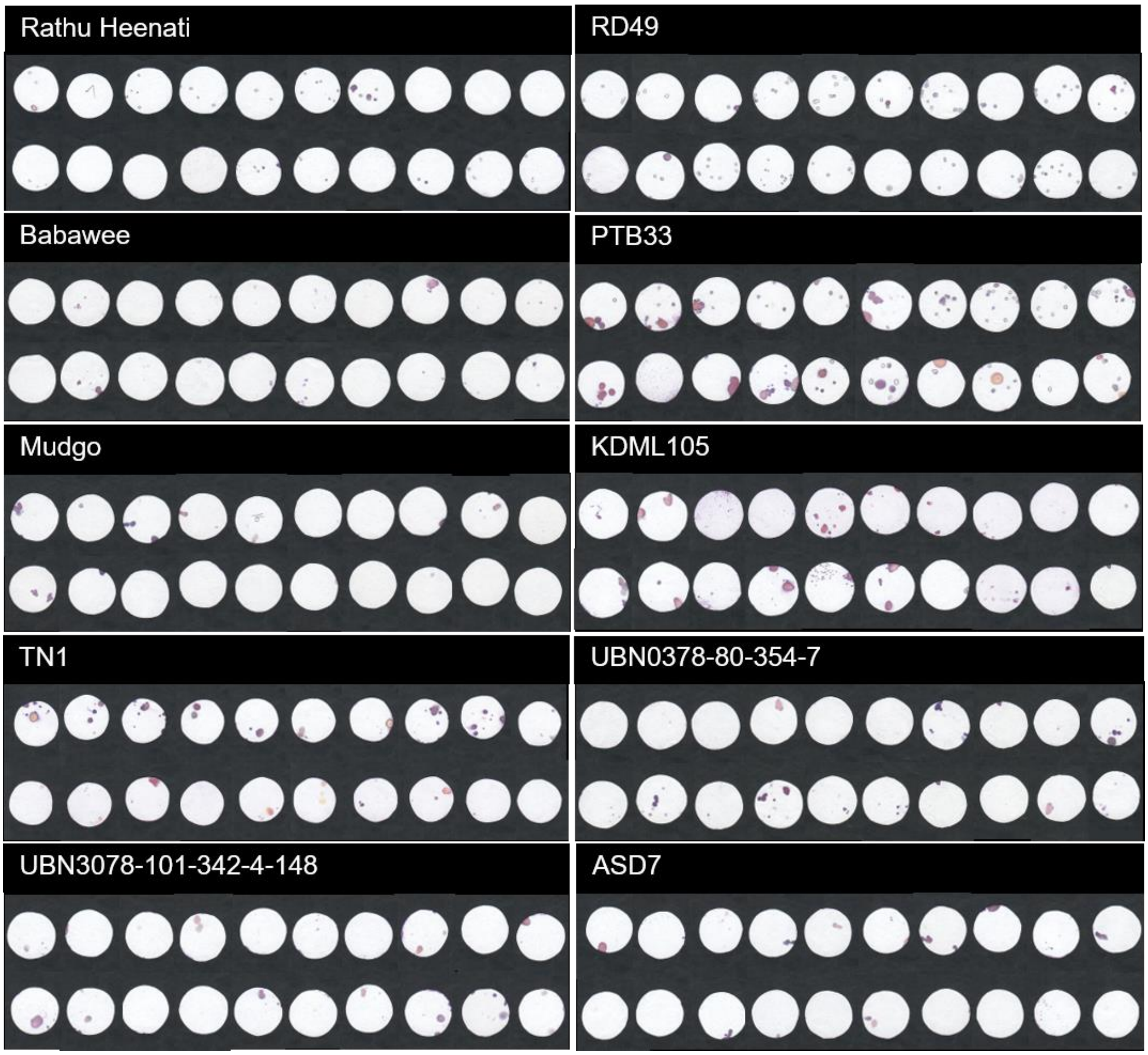

Supplements Methods

Supplemental Figure

Figure S2. (A) Collection of white-backed planthopper (*Sogatella furcifera*) honeydew on Whatman filter paper that was placed at the bottom of insect cages, and (B) representative staining of honeydew spots on filter paper with 0.1% ninhydrin.

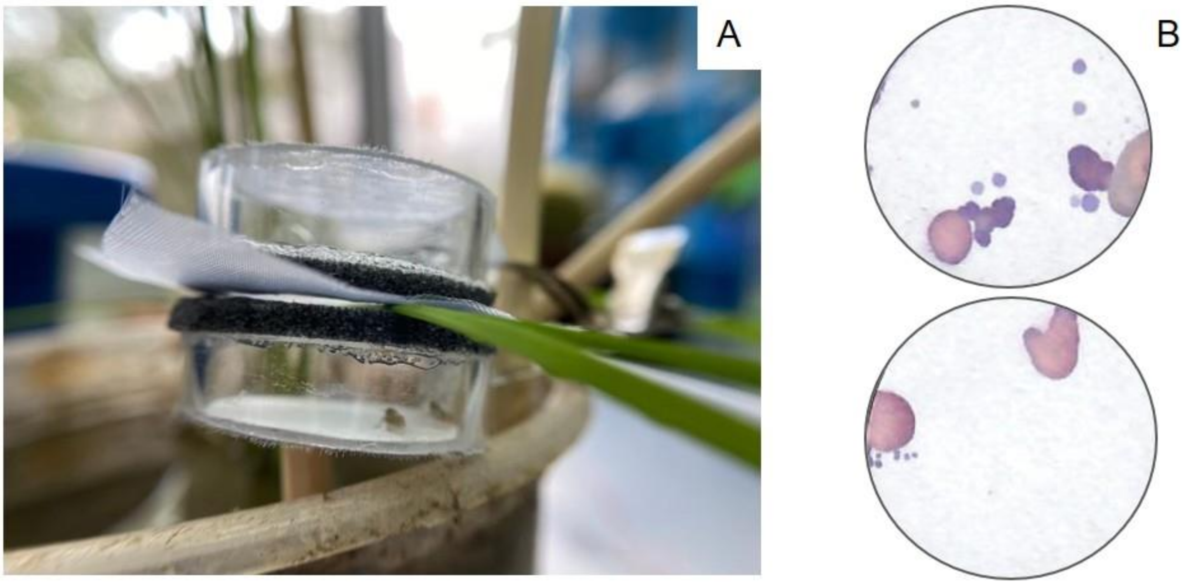

Figure S3. The Rice phenology used for development and survival bioassays. Each seedling was grown individually in a 7–10 cm pot (sterilized substrate), watered daily, and kept free of pesticides.

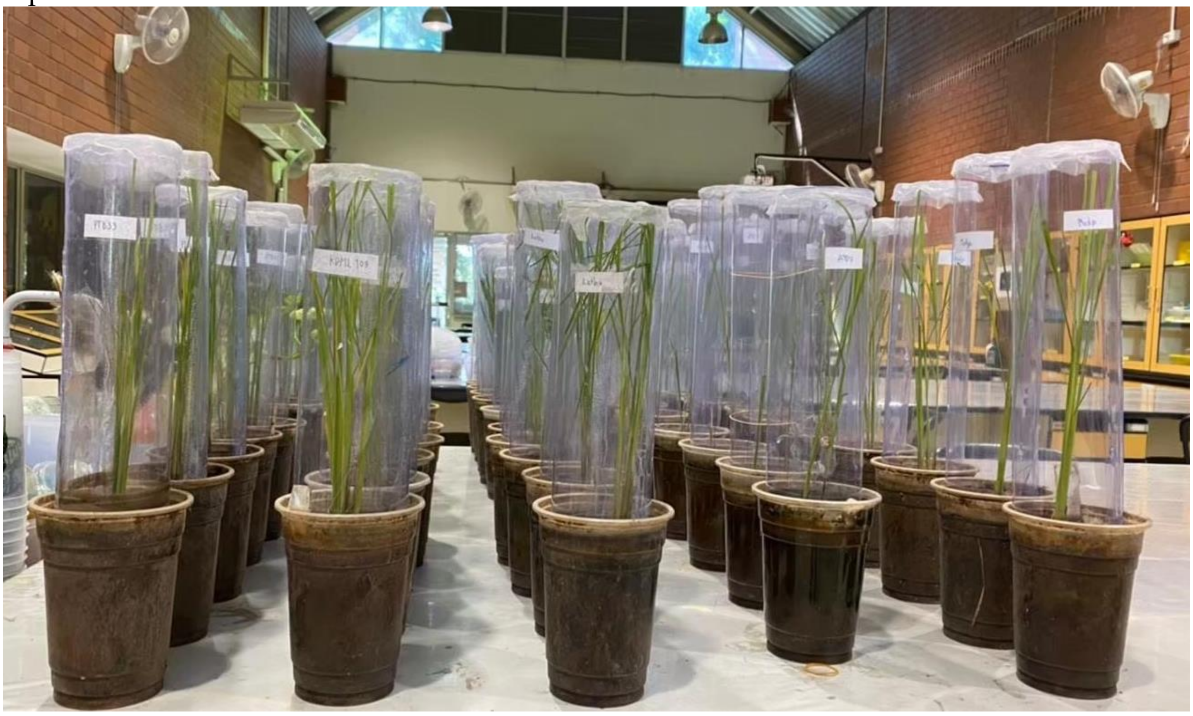

Supplemental Table

Table S1 Sequencing primer and the function of the genes

Genes		Sequences	Functions of the gene
PR10a	Fw	5′CTCAAGTCACACTCGACGGA3′	Classical SA-responsive pathogenesis-related genes. PR1a is a widely accepted molecular marker for SA-mediated systemic acquired resistance (SAR). PR10a, in contrast, has been reported to be strongly induced in resistant rice genotypes and implicated in defense against piercing–sucking insects through ribonuclease activity and interactions with secondary metabolites.
	Rv	5′TTAGGCGTATTCGGCAGGG3′	
PAL1	Fw	5′CGAGGCAATCGACATCCTGA3′	Encoding phenylalanine ammonia lyase, the enzyme that regulates phytoalexin and phenylpropanoid synthesis. PAL1 was included as a key entry enzyme of the phenylpropanoid pathway, which links SA biosynthesis with the production of lignin, phenolics, and other secondary metabolites involved in plant defense.
	Rv	5′GGTCGATCTCCTTGAGCAGG3′	
Chitinase	Fw	5′TACGGCGTGATCACCAACAT3′	The enzyme encoding chitinase digests chitin in the insect exoskeleton. Chitinase was selected as a representative defense enzyme because its induction has been widely associated with antibiosis-based resistance and enhanced defense against insect pests and insect-associated microorganisms.
	Rv	5′TGTTGAACGGCCTCTGGTC3′	
JAR1	Fw	5′AGGATGGGTTGGCTCTAACA3′	A core JA-pathway gene because it catalyzes the conjugation of jasmonic acid to isoleucine (JA-Ile), the biologically active form required for COI1-dependent JA signaling and insect-induced defense responses
	Rv	5′CCTCCCCTATTGGTTTCTCC3′	
JAmyb	Fw	5′CATCTCTGATCACGGCGAGG3′	A JA/SA-responsive transcription factor due to its regulatory role in integrating hormonal crosstalk and modulating the expression of downstream defense-related genes during biotic stress.
	Rv	5′CCAGGCAGATGCTGTGCTAT3′	
Actin-RAc1	Fw	5′GAAGCTGCGGGTATCCATGA3′	The reference gene, due to its stable expression across genotypes and treatments, has been previously validated in rice defense-related gene expression analyses
	Rv	5′GCCAGACTCGTCGTACTCAG3′	
